# Integrating Spatial Heterogeneity into an Analysis between Ecosystem Service Value and Its Driving Factors: A Case Study of Dalian, China

**DOI:** 10.3390/ijerph192417055

**Published:** 2022-12-19

**Authors:** Yuqing Zhao, Zenglin Han, Xiaolu Yan, Xuezhe Wang

**Affiliations:** 1 School of Geography, Liaoning Normal University, Dalian 116029, China; 2 Institute of Marine Sustainable Development, Liaoning Normal University, Dalian 116029, China; 3 Liaoning Feisi Marine Technology Co., Ltd., Jinzhou 121000, China

**Keywords:** ecosystem service value, spatial heterogeneity, driving factors, geo-detector, GWR

## Abstract

Human demand for natural resources has grown, leading to ecological debasement and related ecological system administration. Using Dalian as an example, we estimated the changes in the ecosystem service value (ESV) in 2005 and 2020. We used ArcGIS and spatial statistics to conduct estimations and change analyses of the ESV. Based on the results of the ESV, the geographical detector and geographically weighted regression (GWR) elucidated the contributions of different driving factors of the ESV in a 2 km grid. In summary, these results indicated that: (1) from a holistic perspective, the ESV of Dalian fell by 206.8009 billion CNY over 15 years, and the hot spots were concentrated in both the northern and the western parts, whereas the cold spots were distributed in the central part; (2) according to the results from the geographical detector, land use structure factors influenced the ESV most significantly, followed by socio-economic factors, and the impact of natural factors was relatively small; and (3) according to the results of the GWR, land use structure factors negatively affected the ESV, and the positive impact of the proportion of the natural land area was the most obvious. We conclude that the decline in the ESV reflects the impact of human activities on the ecosystem in the studied landscape. Understanding ESV changes should be made a priority in ecosystem management, and evaluating ESV drivers can contribute to developing land use strategies for policy-making.

## 1. Introduction

Ecosystem services are the connection points between the Earth’s life support and human well-being; the clearer they are defined, the more powerful is the support that can be provided to maintain ecological security [1,2,3]. The ecosystem service value (ESV) plays a pivotal role in quantifying the utility value of the services and support provided by the ecosystem for human survival and development [4]. However, intensified human activities have created an increased demand for land, leading to additional ecological land being occupied, devastated, and transformed into impervious surfaces [5], causing a significant depletion in the supply of ecosystems for the essential functions of human existence, which in turn has caused a drop in the ESV [6]. In this context, clarifying the spatial impact of diverse factors on ESV dynamic changes is of tremendous importance for regional planning and for developing a sustainable human society.

There are two basic methods for studying the ESV and its driving factors. The first method includes traditional quantitative methods, which are often used for investigation in the early stages of research, including regression analyses, logarithmic mean Divisia index, gray correlation degree, and partial least-squares regression models [7,8,9,10,11,12,13]. These traditional methods calculate correlation coefficients by constructing a linear relationship model between the ESV and its driving factors, which can help grasp the driving factors of the ESV for the entire study area. For example, Wang et al. constructed a linear regression model to differentiate the influence of climate change and human activities on the ESV [13]. In another example, Kindu et al. used sensitivity analyses to show estimates of changes in ESVs in response to land use/land cover (LULC) dynamics [10]. While the use of the method in this manner can reveal the impact of LULC changes on ESVs, it can only detect the difference among LULC types in the contributions of the area and cannot unravel complex logical relationships from a spatial point of view; therefore, the method is not suitable for determining the detailed driving mechanism of the ESV, which has obvious spatial heterogeneity characteristics. 

The second approach is working with spatial metering methods, such as the geographically weighted regression (GWR) model, the Bayesian spatiotemporal hierarchy model, and the bivariate spatial autocorrelation analysis, which take spatial heterogeneity of the ESV into account to reveal the impact of driving factors on ESV [14,15,16,17]. For example, Chen et al. first analyzed the spatial interaction between the ESV and economic development with the coupling index method, then found the ESV had significant spatial dependence on economic growth using a bivariate spatial autocorrelation analysis [14]. In another example, Shao et al. added the GWR model and ordinary least-squares regression model when investigating the spatial association between LULC change and the ESV [16]. Although single spatial metering methods can overcome the limitation that the calculations of traditional quantitative methods cannot be expressed spatially, they also make it difficult to demonstrate the role of multiple drivers on the ESV. Therefore, there are still research gaps in the quantitative study of ESV driving factors and their spatial heterogeneity expression.

The above summary shows that a comprehensive analysis of driving factors and the featured approach are both crucial for the study. In terms of the selection of driving factors, current research mainly includes the following direct or indirect aspects. Direct driving factors, which can unequivocally influence the structures, processes, and functions of ecosystems, include land use change [18,19,20,21,22] or climate change and resource consumption [3,13,23]. Indirect driving factors mainly include economic development, urbanization, population agglomeration, and policies. In summary, the driving factors of the ESV are complex and diverse [3,11,15,17,24]. However, finding a reasonable relationship between complex drivers and spatial expression is something that existing studies have not considered. Thus, the integration of these models for understanding drivers is considered to be a path to obtain a comprehensive explanation of ESV changes. At present, the geographical detector and GWR have been widely used to analyze the driving factors of regional economies, eco-environments, and regional development planning. Therefore, it is effective to use geographical detectors, GWR, and other methods that consider geographic characteristics to research the driving factors of ESV change.

This main aim of the study was to explore the feasibility of identifying complex driving factors based on a consideration of the spatial heterogeneity of the ESV. For the practical application of hybrid models, we used the geo-detector and the GWR model to identify the driving factors that change the ESV in Dalian. The results are supportive of ecosystem management and environmental policy-making guidance in Dalian. 

## 2. Materials and Methods

### 2.1. Study Area

**Figure 1:**
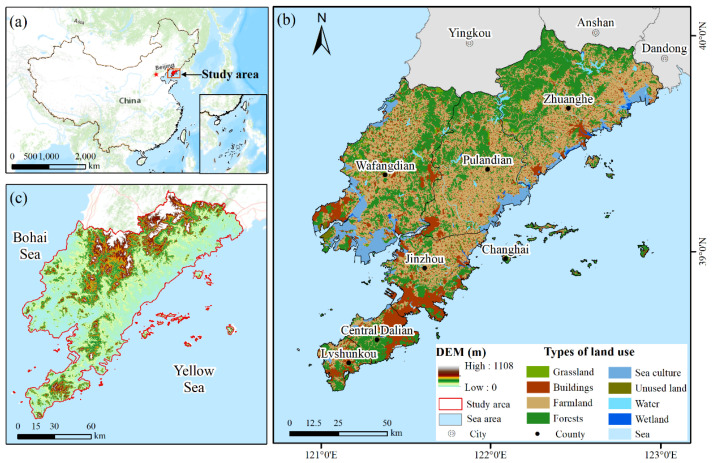
Study area location. (**a**) The location of the study area in China, (**b**) Land use types of Dalian, (**c**) Elevation map of Dalian).

Dalian (38°43′–40°12′ N, 120°58′–123°31′ E) is located in the south of the Liaodong Peninsula, on the coast of the Yellow Sea and Bohai Sea, with the northeast hinterland behind and separated from the Shandong Peninsula (Figure 1). There were 10 districts in the study area. Among them, Zhongshan, Xigang, Shahekou, and Ganjingzi are the central urban areas. The region is characterized by a warm, temperate, semi-humid, continental monsoon climate, with average annual precipitation ranging from 550 to 900 mm, an average annual temperature of approximately 10 °C, and a land area of approximately 12,573.85 km^2^. The coastline of Dalian is 2211 km, accounting for 73% of the total coastline lengths of Liaoning Province and 10% of the coastline of China. The ecosystems include forest, grassland, salt pan, wetland, and ocean. The natural land has been continually destroyed and occupied to meet the human demands of economic development. In March 2021, the Dalian government released its 14th Five-Year Plan, which consolidated the foundation of green development and committed to building an ecologically livable city. Therefore, an in-depth study of the ESV change and its driving forces in Dalian has important practical significance for promoting the construction of Dalian’s ecological civilization and regional sustainable development. 

### 2.2. Data Sources

**Figure 2:**
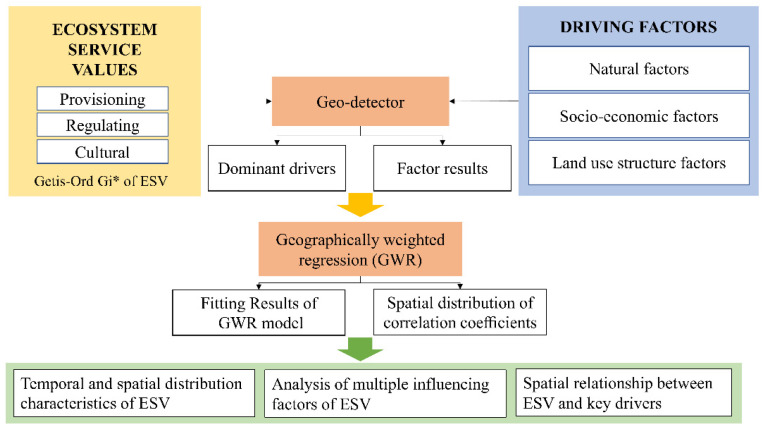
Theoretical and methodological frameworks represented in the flow chart of the work. (Getis–Ord Gi* is a method to explain the spatial distribution of ESV).

This study included the following steps (Figure 2): (1) estimating the ESV from 2005 to 2020; (2) mapping its spatial distribution; and (3) computing the spatial relationship between the ESV and driving forces during the study period. The data used in this study are vector data of land use and driving force data for Dalian from 2005 to 2020. The selection of driving forces is the key to exploring the driving mechanism. The selection of the factor index in this article was made by primarily considering natural, socio-economic, and land use structure factors that may effect changes in the ecosystem functions. Additionally, based on the condition of the natural ecosystem of the study area and the availability of data, 14 types of driving forces were selected, which were divided into 3 categories (Table A1); the categories were further classified and discretized based on the type of data required for subsequent analysis according to the natural breaks method.

### 2.3. Methods

#### 2.3.1. Evaluation of ESV

In this study, we used spatial models and economic methods [25,26] to map the ESV in Dalian. Moreover, to eliminate the impact of price changes in each period, we adopted the constant price in 2015. The value of food production was calculated based on its vendibility [27,28]. The value of the water conservation service was quantified using the reservoir water storage cost method [13] using the water yield model of Integrated Valuation of Ecosystem Services and Trade-offs (InVEST). We applied the carbon tax rate and industrial oxygen production method [13] to quantify the value of carbon sequestration and oxygen release services, both of which were obtained from net primary productivity (NPP) based on the Carnegie–Ames–Stanford approach model (CASA) [29]. The Revised Universal Soil Loss Equation (RUSLE) model, market value method, and shadow project approach were applied to map the value of soil retention services. To calculate the value of habitat support, we used the InVEST habitat quality model [30]. Xie et al. improved Costanza et al.’s research in China based on expert knowledge methods. Their compilation of the Chinese ecosystem service equivalent value per unit area has been widely used. Referring to Xie et al., we mapped the value of landscape aesthetics to land use types [31]. 

(1) Food production

Food production reflects human livelihoods and food security in countries. In this study, food production was quantified as the production of food from agriculture (e.g., grains, fruits, and vegetables), livestock (e.g., meat, eggs, and milk), and fishing (e.g., shrimp, crab, and others). The calculation formula is as follows:

(1) $$S_{f}=\sum Q_{i}\cdot P_{i}\cdot B$$

where Sf is the value of the food production service (CNY/ha/a), Q_i_ represents the annual yield per hectare of the ith food (t/ha/a), P_i_ is the market price of the ith food (CNY/kg), and B is the average profit margin of food sales. The above factors were obtained from the Statistical Yearbook established by the Dalian Municipal Bureau of Statistics.

(2) Carbon sequestration and oxygen release

Based on the photosynthesis and respiration data of the vegetation, 1.63 units of carbon can be sequestered, and 1.2 units of oxygen can be released for every unit of NPP accumulated by the vegetation [32]. The value of carbon sequestration and oxygen release services were calculated using Formulae (2) and (3).

(2) $$S_{c}=\sum NPP\left(x,t\right)\cdot \left(1.63C_{C}+1.2C_{O}\right)$$

(3) $$NPP\left(x,t\right)=APAR\left(x,t\right)\times \varepsilon \left(x,t\right)$$

where S_c_ is the value of carbon sequestration and oxygen release services (CNY/ha/a), C_c_ is the social cost of carbon [33], and C_O_ is the cost of industrial oxygen production. NPP(x, t) is the NPP of position x at time t, APAR(x, t) is the available photosynthetic radiation absorbed by position x at time t (MJ·m^−2^), and ε(x, t) is the light utilization efficiency of pixel x at time t (gC·MJ^−1^). 

(3) Water conservation

Water conservation services play a significant role in controlling soil desertification and reducing soil erosion. We used the InVEST water yield model to quantify the amount of water conservation. The fundamental formulae for the value of water conservation services are as follows:

(4) $$S_{\omega }=\sum \mathrm{WR}\cdot C$$

(5) $$Y_{x}=\left(1-\frac{AET_{x}}{P_{x}}\right)\times P_{x}$$

(6) $$WR=\min\left(1,\frac{249}{velocity}\right)\times \min\left(1,\frac{0.9\times TI}{3}\right)\times \min\left(1,\frac{K_{\mathrm{sat}}}{300}\right)\times Y_{x}$$

where SW is the value of the water conservation service (CNY/ha/a), WR is the amount of water conservation (mm), and C is the reservoir construction cost (CNY/m^3^). Y_x_ is the annual yield at pixel x (mm), P_x_ is the average annual precipitation at pixel x (mm), and AET_x_ is the actual annual evapotranspiration at pixel x (mm). The above factors of the InVEST water yield model are ensured by referring to the InVEST guidebook. The velocity is the coefficient, TI represents the terrain index, and Ksat means the soil saturated water conductivity (cm/d).

(4) Soil retention

The RUSLE model was used to estimate the potential and actual soil erosion in the study area. The spread between them is the amount of soil retention [34]. The value of soil retention services due to vegetation was quantified based on the role of vegetation in reducing land loss and sediment accumulation. The formulae are as follows:

(7) $$S_{s}=\sum S\cdot 24\%\cdot C÷\rho +\sum S\cdot F÷\rho ÷d$$

(8) $$S=R\cdot K\cdot LS\left(1-C\cdot P\right)$$

where S_s_ is the value of soil retention services (CNY/ha/a), S is the amount of soil retention (t/ha/a), and 24% represents the ratio of mud and sand accumulated in reservoirs, rivers, and lakes in China [35]. C is the reservoir construction cost (CNY/m^3^), ρ is the soil bulk density (g/cm^3^), d is the average soil thickness (m), and F is the average forestry income (yuan/ha). R represents the erosion factor of rainfall, K represents soil erodibility, LS represents slope length and slope, C represents vegetation cover and crop management factors, and P stands for soil and water conservation measures. The process for calculating R, K, LS, C, and P factors is described in Remortel et al., Williams and Arnold, and Wischmeier and Smith [36,37,38].

(5) Habitat support

Habitat support is the potential for an ecosystem to create conditions for species survival and reproduction, which is reflected in the habitat quality index [39]. The distribution of habitat quality was obtained using a combination of landscape type sensitivity and external threat intensity based on InVEST habitat quality. Given the primary protected animals in Dalian (e.g., migratory birds, vipers, and harbor seals), we regarded the land use types of buildings, farmland, and aquaculture as external threat factors.

(9) $$S_{h}=\frac{Q_{\mathrm{xj}}}{\bar{Q}}\cdot V_{B}$$

(10) $$Q_{\mathrm{xj}}{=H}_{j}\left[1-\left(\frac{D_{xj}{}^{z}}{D_{xj}{}^{z}+k^{z}}\right)\right]$$

(11) $$V_{B}=\sum \frac{1}{7}\cdot Aj\cdot \frac{Sj}{S}\cdot Q\cdot P_{f}$$

where S_h_ is the value of habitat support services (CNY/ha/a), Q_xj_ is the habitat quality of grid x in land use type J, $\bar{Q}$ is the average annual habitat quality index, and VB is the baseline value of biodiversity (CNY/ha/a). H_j_ is the habitat suitability of land use type J, D_xj_ is the stress level of grid x in land use type j, K is the semi-satiety constant and is usually half of the maximum value of D_xj_, and Z is the conversion factor. The ratio 1/7 represents the equivalent coefficient of the ESV, A_j_ represents the unit area value equivalent factor for the habitat support service provided by land use type j, Q is the average grain yield per unit area (kg/ha), and P_f_ is the average market price of grain in Dalian in 2015 (CNY/kg).

(6) Landscape aesthetics

Landscape aesthetics are embodied in the evaluation of landscapes with recreational, cultural, and artistic value. In addition, considering that there is some error in representing regional characteristics with a national parameter table, it is necessary to correct this. Since tourism can indirectly reflect landscape aesthetics, the service value is revised according to tourism income.

(12) $$S_{l}=V_{l}\cdot \frac{t}{T}$$

(13) $$V_{l}=\sum \frac{1}{7}\cdot Lj\cdot \frac{Sj}{S}\cdot Q\cdot P_{f}$$

where S_l_ is the value of landscape aesthetic service (CNY/ha/a), t is the average tourism income of Dalian (CNY/ha), and T is the national average tourism revenue (CNY/ha). Additionally, 1/7, S_j_, S, Q, and P_f_ are the same as above. L_j_ is the unit area value equivalent factor for the landscape aesthetic service provided by land use type j. 

#### 2.3.2. Calculation of Driving Factors

(1) Human active index

The human active index (HAI) has spatial variability, reflecting the influence of anthropogenic activities on land use and landscape composition changes. As a result of anthropogenic activities, the original natural characteristics of the landscape components are decreasing, and different types of landscape components represent different characteristics of anthropogenic activities or exploitation intensity. The following expression is used to calculate HAI:

(14) $$HAI=\sum_{l=1}^{N}A_{i}P_{i}/TA$$

where HAI is the anthropogenic influence index, N is the number of landscape types, A is the total landscape area, P_i_ is the anthropogenic influence intensity parameter reflected by the landscape component, and TA is the total landscape component area. P_i_ was determined using the Lohani checklist method, the Leopold matrix method, and the Delphi method [40,41,42,43]. To reduce the error, we took the average of the three coefficients as the coefficient (Table 1).

(2) Land use intensity index

The land use intensity index (LUI) uses a discontinuous function to express land use intensity [44], reflecting the degree of human development and utilization of land. Li et al. hypothesized that different land use types can be attributed to LUI with different utilization intensities [42]. Mudflats and unused land are classified as LUI-1; forest, grassland, and open water as LUI-2; and aquaculture as LUI-3. The agricultural land type is LUI-4, and construction land is LUI-5. Therefore, this study divides the land use types in the study area into five types corresponding to different LUIs (Table 2).

In the actual state, land use types are randomly combined in the same area, each with a different area weight, contributing to the local LUI according to their weights. Thus, the comprehensive quantitative indicators of LUI can be mathematically synthesized. The magnitude of their values integrally reflects the degree of land use:

(15) $$L=100\times (\sum_{i=1}^{n}P_{i}\times Q_{i})$$

where L is the composite index of LUI in the study area, P_i_ is the level LUI in the study area (i = 1, 2, 3, 4, and 5), Q_i_ is the percentage of the area occupied by the ith level landuse type in the study area, and n is the number of LUI classifications in the study area.

#### 2.3.3. GIS Analysis and Spatial Statistics

Presently, when most scholars research ESV driving factors, they primarily select nations [22], provinces/states [24], cities, or particular regions [18,19,20] as their research objects. As current research lacks grids and other small-scale studies, the ESV is spatially affected by different types of land and is diverse within a space. The methodological approach in this study is a primarily based on the grid method to process the ESV and its driving factors in space. Thus, factors are implemented in a smaller space, so the differences between different regions can be studied.

(1) Hot and cold spot analysis

A hot spot is a location or a small area within an identifiable boundary showing the concentration of incidents. This tool works by looking at each piece of data in the data environment of neighboring cells. To be a statistically significant hotspot, not only should the data itself have high values, but there also needs to be a certain number of clusters of high-value data around it. The Getis–Ord Gi* is expressed as follows [45,46]:

(16) $$G_{i}{}^{*}\left(d\right)=\frac{\sum_{i=1}^{n}w_{ij}(d)x_{j}}{\sum_{j=1}^{n}x_{j}}$$

where x_j_ represents the observation in unit j, w_ij_(d) represents the spatial weights between spatial units i and j, and n represents the number of units.

(2) Geographical detector method

A set of statistical methods used by geographical detectors can explore the consistency in spatial distribution between dependent and explanatory variables through spatial dissimilarity and reveal the driving forces behind the spatial distribution characteristics of the dependent variable [47,48]. The value of the q-statistic in the factor detector is used to measure the explanatory power of the explanatory variables for the spatial divergence of the dependent variable, detecting the extent to which a given driver X can be used to explain the spatially divergent characteristics of Y. The degree of spatial association was measured using the q-statistic [49]:

(17) $$q=1-\frac{1}{N\sigma^{2}}\sum_{h=1}^{L}N_{h}\sigma_{h}{}^{2}$$

where q is the determination power of X to the ESV; n is the number of sample units; Nh and N are the variances of the ESV of region h and Dalian, respectively; and σ_h_^2^ and σ^2^ are the variances of the ESV of sub-region h and the whole region, respectively. L denotes the total number of regions h.

(3) Geographically weighted regression

Due to the spatial complexity, autocorrelation, and variability in the ESV data, when exploring the influence of explanatory variables on dependent variables, their expression may be different in different regions. Therefore, based on the use of a geo-detector to determine the critical factors of the ESV, this study used GWR to analyze the spatial differentiation of driving factors to explore how each driving factor serves the ESV in different regions. The GWR model was expressed as follows:

(18) $$y_{i}=\beta_{0}(u_{i},v_{i})+\sum_{k=1}^{n}\beta_{k}(u_{i},v_{i})x_{\mathrm{ik}}+\varepsilon_{i}$$

where (u_i_,v_i_) are the spatial coordinates of the sample point i, β_k_(u_i_,ν_i_) is the value of the continuous function β_k_(u,ν) at the point i, x_ik_ is the explanation variable x_k_ in the location (u_i_, v_i_) and ε_i_ is the error term. The GWR model constructed has the highest model accuracy by drawing on the research results in the calculation and, through practice, the fixed Gaussian function is determined as the weight function, the bandwidth is determined by the AIC method, and the GWR4 is used for the regression calculation.

## 3. Results

### 3.1. Variability of the ESV

**Figure 3:**
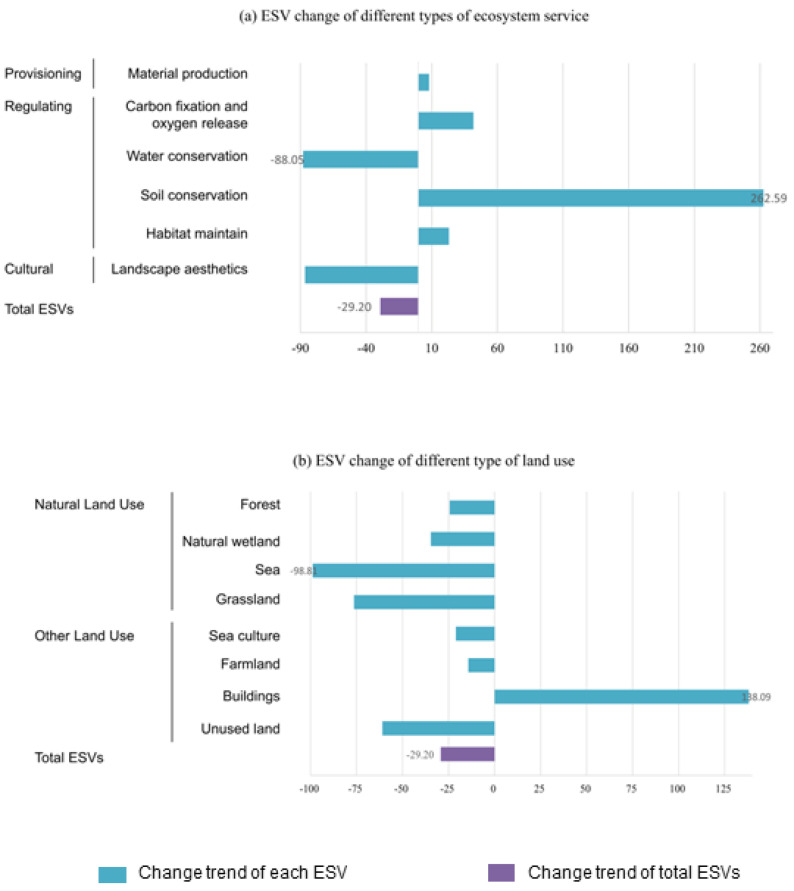
Change trends of ESVs provided by Dalian during the whole study period (2005–2020). The names of different constituent parts and categories are noted on the left side. The particular change rate is marked in the numeric value. (**a**) ESV change of different types of ecosystem service. (**b**) ESV change of different type of land use.

Accounting for ESVs in 2005 and 2020 in Dalian according to Equations (1)–(13), the value provided by each ecosystem service type and different ecosystem types was summarized and analyzed. These results are shown in Figure 3 and Table A2.

In 2005, the total ESV in Dalian was 708.2845 billion CNY. The majority was constituted by forests (336.2547 billion CNY), accounting for 47.47% of the total ESV. Farmland, sea culture, and sea accounted for 21.91%, 10.56%, and 9.48% of the total ESV, respectively. The other four ecosystems (wetland, grassland, buildings, and unused land) contribute to the remaining 10.58% of the ESV. In 2020, the total ESV in Dalian was 501.4836 billion CNY. The majority still constituted forests (254.2054 billion CNY), accounting for 50.69% of the total ESV. Farmland and sea culture accounted for 26.52% and 11.77% of the total ESV, respectively. The ESV contributions of the other five ecosystems were lower than 10%. The aggregated ESV for forest, sea culture, and farmland accounted for more than 80% of the total ESV in 2005 and 2020, suggesting that these ecosystem types provide most of the ecosystem services in Dalian.

Between 2005 and 2020, the total ESV loss was 206.8009 billion CNY, representing a decrease of 29.20%. The rate of decrease was evident during the last 15 years of the study period. From the perspective of ecosystem service function, although the proportion of the ESV provided by various ecosystem service types changed in different periods, the composition structure and contribution rate were similar. From 2005 to 2020, the ESV of water conservation and landscape aesthetics decreased sharply by 219.37 billion CNY and 112.69 billion CNY, respectively. The decrease in the ESV of water conservation and landscape aesthetics caused by it is mainly provided by forest and farmland; these two land use types have high-value contribution rates and cover a large area. The values of the other ecosystem service function types increased at different rates. Affected by the heavy rainfall, the reduction in the value of water conservation was the largest.

From the ESV change of different land use types, the ESV of buildings showed an upward change. In contrast, the ESV of the other land use types declined. This reduction is most apparent in the sea and was caused by a sharp decrease in the ocean area. During the study period, the sea area decreased by 329.59 km^2^, and the land use of sea culture occupied a large area of the sea. Based on this phenomenon, more attention needs to be paid to sea management.

### 3.2. Spatiotemporal Distribution Characteristics of ESV

**Figure 4:**
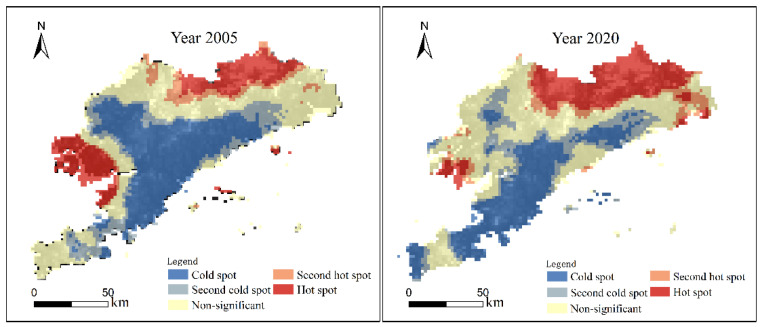
Getis–Ord Gi* spatial distribution of Dalian’s ESV per unit area in 2005 (**left**) and 2020 (**right**).

In this study, Getis–Ord Gi* in ArcGIS software was used to describe the spatial distribution features of the ESV per unit area in Dalian. The grid-scale used is 2 km per unit, mainly set based on the data resolution and the area of the administrative unit in Dalian. The two images below represent a significant observation of hot and cold spots (Figure 4).

From a temporal and spatial perspective, there were similar distribution characteristics between 2005 and 2020: the hot spots were distributed in both the northern and western parts, and the cold spots were distributed in the middle part. The spatial variability in the distribution pattern of the ESV hot spot and cold spot is mainly in the following aspects: (1) The area with a cold spot extends toward the southern direction. Comparing the results over two years, the areas where cold spots were significantly reduced were located in Wafangdian and Zhuanghe. There was a dramatic increase in the cold spot area of the ESV in the southern region. (2) The hot spot spatial distribution slightly decreased during the study period, mainly concentrated in Dalian’s northern and southwestern regions.

### 3.3. Analysis of Driving Factors for Variation in the ESV

A geo-detector can accurately analyze the determination power of the driving forces. In this study, the raster data of driving forces and the ESV were resampled at 2 km. The 2005 data were divided into 3040 units. The 2020 data were divided into 2810 units. All data were divided into nine classes based on the reclass tool in ArcGIS. The classification information of the raster datum was extracted for each sample location with the ESV, following which they were input into the geo-detector.

As shown in Table 3 the relative importance of driving forces on the ESV change in 2005 was as follows: NDVI (0.2763) > LUI (0.2482) > HAI (0.2252) > POP (0.1908) > GDP (0.1786). The results indicate that NDVI, which displayed the highest q-statistic, predominantly explains the change in the ESV, followed by LUI and HAI. In 2020, the top five individual driving forces associated with the ESV change were as follows: LUI (0.4677) > HAI (0.4251) > Proportion of natural land area (0.3928) > DEM (0.2669) > SLOPE (0.1896). These results indicate that LUI, which displayed the highest q-statistic, predominantly explains the change in the ESV in 2020, followed by the HAI and proportion of natural land area. Overall, we observe that human activity led to changes in land use, which directly affected the ESV of the whole area. The impact of natural factors on the ESV was the next strongest, with socio-economic factors being the weakest.

In summary, comparing results from 2005 and 2020, we observed that natural and socio-economic factors and land use structure have varying degrees of impact on ecosystem services. We focused on five dominant drivers based on the results of the geo-detector, which provided scientific support for subsequent research.

### 3.4. Spatial Relationship between the ESV and Key Drivers

**Figure 5:**
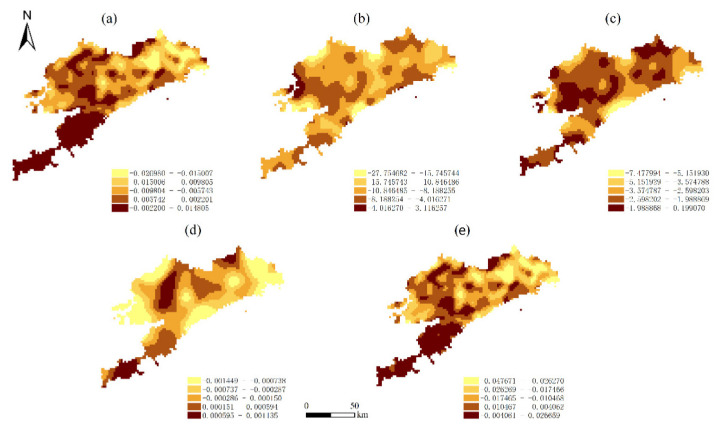
Spatial distribution of correlation coefficients between the ESV per unit area and its driving forces in the GWR model in 2005: (**a**) GDP per unit area; (**b**) HAI; (**c**) LUI; (**d**) NDVI; (**e**) POP per unit area.

**Figure 6:**
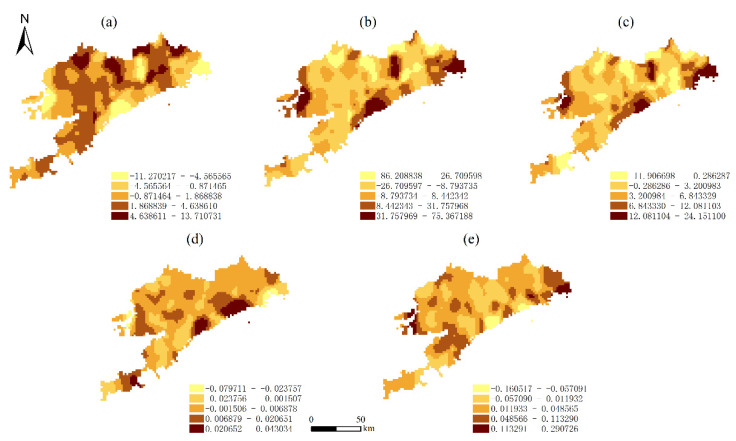
Spatial distribution of correlation coefficients between the ESV per unit area and its driving forces in the GWR model in 2020: (**a**) LUI; (**b**) HAI; (**c**) proportion of natural land area; (**d**) DEM; (**e**) slope.

To explore the differential characteristics of the spatial distribution of the factors influencing the ESV, we applied the GWR model to estimate the spatial divergence parameters of the factors influencing ESV in Dalian; the ESV was calculated separately from the five explanatory variables in the calculations. The fitting results are listed in Table 4, Figure 5 and Figure 6 present the contributions of the five key driving forces to the changes in the ESV per unit area over the study period.

In 2005, positive coefficients accounted for 38.55% of the impact of NDVI on the ESV, and negative coefficients accounted for 61.45%. In 2005, the region of negative coefficients had more than 90% of the impact of the remaining four driving forces on the ESV, which had an apparent adverse effect. This indicates that frequent human activity may cause a reduction in the ESV. In 2020, the impact of HAI and LUI on the ESV, the negative coefficients, accounted for 67.4% and 28.7%, respectively. Regarding the impact of DEM and slope on the ESV, the negative coefficients, which belong to the socio-economic category, accounted for 31.4% and 17.9%, respectively. Regarding the impact of the proportion of natural land area, the positive coefficient accounted for 85.5% of the ESV. Meanwhile, although the proportion of natural land area change increased, the growth rate of the ESV also increased.

The negative coefficients of the two driving forces (LUI and HAI) on the ESV exhibited a slight decrease, respectively, during the entire 2005–2020 period, which partly contributed to the decreasing trend of the ESV. The negatively correlated regions were mainly distributed in the northeastern area of Dalian. These results indicate that the exploitation of the ecosystem in the region should be restrained. In general terms, the effects of socio-economic factors on the ESV were mainly adverse, which suggests that the exploitation of the ecosystem may simultaneously cause a decrease in the ESV.

## 4. Discussion

### 4.1. Exploring Driving Factors for the ESV at Multiple Methods

ESV changes are not caused by a single factor, but by a combination of factors. Generally, natural factors, socio-economic factors, and land use factors are three common aspects [13,50,51]. Compared to the use of traditional single methods, the combination of the geo-detector and the GWR model shows two outstanding advantages: (1) it takes into account as comprehensive a set of driving factors as possible [17]; and (2) it realizes the integrated analysis of the spatiotemporal pattern, which is helpful for the adequate protection of the ecosystem [52].

During the process of method combination, there are different data sources for driving factors; it is essential to reduce the calculation error caused by the data and accurately determine the degree of their effect on the ESV change. Different data sources for impact factors may have introduced errors into the calculation results when analyzing ESV driving factors. Thus, we selected a 2 km grid as the basis for our analysis at the micro-scale, which offset the error caused by the data. After the data were grided, we used geo-detector to identify the critical drivers of ESV change. According to the results of the geo-detector, five factors had a noticeable impact on the ESV of Dalian in 2005 and 2020. To better show the spatial distribution of the driving factors, we selected the GWR model to improve the analysis of the driving factors. We found that the relevant ecological management policies required for different regions should be different.

Based on the calculation results for the maintenance and improvement of Dalian’s ESV, functions can be divided according to the various roles played by other regions in economic development and ecosystem service provision. For leading ESV providers (Zhuanghe, Wafangdian, and Pulandian), their development should not be based on GDP growth as the main criterion. Nevertheless, strengthening the protection of the ecosystem should be supported. For areas with frequent economic activities (Jinzhou and core city), they should limit the unrestricted damage to the ecosystem caused by economic activities in the area and increase urban green space to ensure that the ecosystem services are available to residents. For Lushunkou and Changhai, ecological protection and economic development should be guaranteed together. Overall, the combination of these two methods can provide new research ideas for exploring ESV driving factors.

### 4.2. Implication of the Relationship between Diverse Factors and the ESV

This study reveals the characteristics of the spatial and temporal changes of the ESV in Dalian during the 15 years from 2005 to 2020. The driving factors that caused these changes were analyzed. According to the calculation results of the geo-detector, we found that land use change caused by human activities has the most significant impact on the ESV. Moreover, population accumulation and economic development have a particular impact on the ESV.

From 2005 to 2020, Dalian’s economic development relied primarily on secondary industries, such as petrochemicals and manufacturing. Additionally, tertiary industries, such as services, information, and finance, have gradually developed and expanded. Simultaneously, Dalian has been actively constructing industrial parks, such as coastal industrial zones and chemical industrial parks, to expand industrial development space, thereby forming multiple economic growth points and driving economic development of the region from point to point. As a coastal city, Dalian is committed to promoting high-quality development of the marine economy. As such, marine emerging industries and marine service industries have been selected to drive development. Hence, it is inevitable that higher requirements for land use will be put forth when promoting economic development in an all-around manner. For example, the development and expansion of industrial parks will inevitably cause damage to the ecological land around parks, such as forests or grasslands, and affect the ecosystem services they provide. The Changxing Island industrial park was constructed in 2005. It can be observed that the conflict between the region’s economic development and the ESV was more serious in 2005 than in 2020. Hence, it is necessary to increase attention to ecosystems and nature to achieve sustainable green development in the true sense when pursuing rapid economic development.

The continuous growth of the population has also invisibly increased the pressure on the ecosystem, which impacts the ESV. The total population of Dalian has increased by 5% over 15 years, with the population growth rate of the municipal district being the largest at 11.8%. Meanwhile, the urban land area of the municipal district increased by 153.37 km^2^, which means that almost the same amount of ecological land has been destroyed. From the perspective of different regions, the conflict between Zhuanghe’s population and the ESV is the most obvious. Zhuanghe is located in northeast Dalian, covering 387,400 hm^2^, of which 159,600 hm^2^ consists of forest and grassland, accounting for 41.2%. Moreover, Zhuanghe has abundant freshwater resources, including 365 large and small rivers and 45 reservoirs in the territory, with a total annual freshwater volume of 1.88 billion cubic meters. Based on abundant natural resources, it can be determined that Zhuanghe is the main area provided by the ESV in Dalian. Therefore, there is a significant conflict between population growth and the ESV in this area, and the contradiction is prominent.

For different functional subjects and regions of different stages of development, the key factors that cause ESV changes are different. In the early stage of regional economic development, due to the increase in land demand from human activities, the rise of economic activities puts pressure on natural ecosystems, leading to conflicts between humans and nature. At this stage, GDP has a noticeable impact on the ESV. The same situation occurs in China’s Bohai Sea, Ganjiang upstream watershed, and other areas with better natural ecological conditions [7,12,53]. With the continuous occurrence of changes, such as population concentration and increased economic activity, under the influence of changes in people’s consciousness and policy guidance, such as the implementation of the sustainable development concept, the conflict between humans and nature is no longer single and directly manifests itself between GDP and the ESV. The driving factors of the ESV become diverse, despite having some common ground. The allure of better-developed areas for talents and enterprises is enormous, the pressure on infrastructures, such as road construction and urban expansion, has increased at the same time, and land use and population have become the usual key factors affecting the ESV [3,11,16,54].

### 4.3. Contributions

The work reported here is significant both in its innovative use of methods and the practical use of this innovation. The main contribution of this article is the increased attention paid to the study of spatial characteristics of ESV driving factors. The study reported in this article shows that the combination of the geo-detector and the GWR model is sound, and the method is effective to understand how to analyze the impact of diverse driving factors on the ESV.

The combination of the geo-detector and the GWR model is significantly different from the existing methods (such as traditional statistic methods and gray correlation analysis) in that it uses the respective strengths to identify the impact of driving factors on the ESV in different regions. Meanwhile, the findings enrich our understanding of the declining ESV at the grid scale, verify the feasibility of using the two methods together, and provide an important reference for maintaining sustainable development of the ecosystem.

## 5. Conclusions

This article provides new ideas for research into the ESV and its driving factors. This study estimated the ESV of Dalian in 2005 and 2020 based on spatial models and economic methods. The decreased rate of ESV was evident during the last 15 years of the study period in Dalian. Moreover, we used hot and cold spot analysis in ArcGIS software to describe the spatial distribution features of the ESV per unit area in Dalian. We further focused on exploring the effects of various ESV drivers. In the investigation of the role of driving factors, we used the geo-detector and the GWR model to analyze the ESV changes in Dalian by combining 14 driving factors in 3 aspects: natural, socio-economic, and land use structure. Comparing the three driving factors, the most influential type was socio-economic, followed by natural factors, with land use having minimal impact on the ESV. In addition, diversity and spatial heterogeneity emerged as the drivers of the ESV. The response of the ESV to changes in the same impact factors varied across regions and has prominent agglomeration characteristics. Through various research methods and accounting perspectives to calculate the ESV based on safeguarding stakeholders’ interests and enhancing sustainable human well-being, it will be beneficial to clarify the partition of ecosystem functions and guide targeted land use in policy-making.

## Figures and Tables

**Table 1:** Human activity influence intensity coefficients of different landscape components in Dalian.

	Forest	Dry Farm	Paddy Field	Grassland	Building	Wetland	Salt Pan	Other Land Use	Water
Lohani	0.12	0.57	0.55	0.09	0.96	0.15	0.08	0.11	0.10
Leopold	0.11	0.58	0.57	0.08	0.94	0.13	0.09	0.06	0.14
Delphi	0.13	0.62	0.65	0.10	0.91	0.14	0.07	0.07	0.12
Average	0.12	0.59	0.59	0.09	0.94	0.14	0.08	0.08	0.12

**Table 2:** Division of land use intensity in Dalian.

LUI Types	Land Use Types	LUI Types	Land Use Types
LUI-1	Mudflat	LUI-2	Shrubbery
	Other land use	LUI-3	Fish farming
	Moor	LUI-4	Dry farm
LUI-2	Ocean		Paddy field
	Water		Salt pan
	Forest	LUI-5	Building
	Grassland		

**Table 3:** Factor results for ESV and driving forces in Dalian.

		NDVI	LUI	HAI	POP	GDP	Proportion of Natural Land Area	Urbanization Rate
2005	*q* statistics	0.2763	0.2482	0.2252	0.1908	0.1786	0.1590	0.0856
	P	0.000	0.000	0.000	0.000	0.000	0.000	0.000
2020	*q* statistics	0.1312	0.4677	0.4251	0.1278	0.1489	0.3928	0.0997
	P	0.000	0.000	0.000	0.000	0.000	0.000	0.000
		SLOPE	DEM	SHDI	PD	≥10 °C Cumulative temperature	PRE	Soil erosion
2005	*q* statistics	0.0681	0.0623	0.0597	0.0523	0.0267	0.0143	0.0078
	P	0.000	0.000	0.000	0.000	0.000	0.000	0.931
2020	*q* statistics	0.1896	0.2669	0.0185	0.0020	0.0665	0.0541	0.1084
	P	0.000	0.000	0.000	0.000	0.025	0.000	0.000

Abbreviations: normalized difference vegetation index (NDVI), land use intensity (LUI), human activity index (HAI), population per unit area (POP), gross domestic production per unit area (GDP), digital elevation model (DEM), Shannon’s diversity index (SHDI), patch density (PD), precipitation(PRE). Interpretation of Indicators: SLOPE means slope of terrain.

**Table 4:** Results of theGWR model of the ESV and key factors in Dalian.

Diagnostic Information	2005	2020
Bandwidth size	118.501637	116.070957
Residual sum of squares	18,087.061042	5086.711880
Unbiased sigma estimate	2.616989	1.443917
AICc	14,705.267611	10,263.690133
R square	0.415633	0.590915
Adjusted R square	0.327750	0.528816

Abbreviations: Corrected Akaike information criterion (AICc)

## Data Availability

All data and code are available via the corresponding author.

## Appendix A

**Table A1:** Data sources and processing.

Category	Driving Forces	Computing Method of Data	Data Sources
Natural factors	Digital elevation model	-	http://www.gscloud.cn/
	Slope	Extracted by DEM data with ArcGIS slope tool.	http://www.gscloud.cn/
	Normalized difference vegetation index	-	https://www.nasa.gov
	≥10 °C Cumulative temperature	Calculation of inverse distance weight space interpolation used temperature data measured by 17 meteorological stations located around Dalian.	http://data.cma.cn
	Precipitation per year	Calculation of inverse distance weight space interpolation used precipitation data measured by 17 meteorological stations located around Dalian.	http://data.cma.cn
	Soil erosion	-	-
Socio-economic factors	Gross domestic production per unit area	-	http://www.resdc.cn/
	Population per unit area	-	http://www.resdc.cn/
	Urbanization rate	Calculation by DMSP nighttime light image.	http://www.resdc.cn/
	Human active index	Calculation by land use vector data in Dalian.	-
Land use structure factors	Land use intensity	Calculation by land use vector data in Dalian.	-
	The proportion of natural land area	Calculation by land use vector data in Dalian.	-
	Shannon’s diversity index	Calculation by land use vector data in Dalian using a moving window of FRAGSTATS.	-
	Patch density	Calculation by land use vector data in Dalian using a moving window of FRAGSTATS.	-

Note: DEM and Slope accessed on 13 February 2021, NDVI accessed on 5 March 2021, ≥10 °C Cumulative temperature and PRE accessed on 12 March 2021, GDP, POP and DMSP nighttime light image accessed on 8 June 2021.

**Table A2:** Temporal and spatial changes in ecological service value in Dalian from 2005 to 2020 (billion CNY, %).

		Forest	Sea Culture	Wetland	Sea	Grassland	Farmland	Buildings	Unused Land	Total	ESV Change	Change Rate (%)
Material production	2005	0.0065	42.8168	0.1508	26.0804	0.6316	42.9306	0.0000	0.0000	112.6167	8.9590	7.96
	2020	16.6685	57.5037	0.4189	0.6796	0.7094	45.4689	0.0000	0.1267	121.5757		
Carbon fixation and oxygen release	2005	95.2435	0.7008	2.9127	0.0022	7.0191	33.2491	6.1475	0.7275	146.0024	61.1746	41.90
	2020	109.4548	0.9957	8.2204	0.0065	2.9108	67.0139	17.8513	0.7235	207.1770		
Water conservation	2005	123.7115	28.1347	11.8123	19.8398	10.0058	54.2323	0.0000	1.3996	249.136	−219.3689	−88.05
	2020	19.0810	0.0051	0.0658	0.0001	0.6572	9.9161	0.0000	0.0420	29.7671		
Soil conservation	2005	8.7877	0.2199	0.2228	0.0428	0.7589	2.8168	3.3311	0.0390	16.219	42.5897	262.59
	2020	43.3939	0.1551	0.8857	0.0047	0.5824	8.9865	4.7156	0.0847	58.8087		
Habitat maintenance	2005	33.4381	1.3802	2.6368	2.2877	2.7580	11.2914	0.0000	0.3576	54.1498	12.5039	23.14
	2020	52.8476	0.2482	11.8171	0.0803	0.6927	0.9839	0.0000	0.0109	66.6807		
Landscape aesthetics	2005	75.0674	1.5151	20.7595	18.9134	3.1959	10.6951	0.0000	0.0139	130.1603	−112.6859	−86.58
	2020	12.7597	0.1167	3.7518	0.0290	0.2123	0.6023	0.0000	0.0027	17.4744		
Total	2005	336.2547	74.7676	38.4951	67.1664	24.3693	155.2153	9.4785	2.5376	708.2845	−206.8099	−29.20
	2020	254.2054	59.0245	25.1598	0.8001	5.7648	132.9716	22.5670	0.9905	501.4836		
ESV change		−82.0493	−15.7431	−13.3353	−66.3663	−18.6045	−22.2437	13.0885	−1.5471	-	-	-
Change rate (%)		−0.2440	−0.2106	−0.3464	−0.9881	−0.7634	−0.1433	1.3809	−0.6097	-	-	-
